# Diagnosis and rescue of malignant hyperthermia induced by anesthesia during radical surgery in a cervical cancer patient using the National Remote Emergency System: A case report

**DOI:** 10.1097/MD.0000000000037699

**Published:** 2024-04-19

**Authors:** Yang Xiao, Rou Yu, Juan Gu

**Affiliations:** a Department of Anesthesiology, West China Second University Hospital, Sichuan University, Chengdu, China; b Key Laboratory of Birth Defects and Related Diseases of Women and Children (Sichuan University), Ministry of Education, Chengdu, China.

**Keywords:** Case report, diagnosis, general anesthesia, malignant hyperthermia, National Remote Emergency System

## Abstract

**Rationale:**

Malignant hyperthermia (MH) is a rare yet serious medical complication that typically arises following general anesthesia or the administration of specific anesthetics. Due to the infrequency of MH, anesthesiologists often lack sufficient expertise in identifying and managing it, leading to misdiagnosis and inappropriate treatment. There is an urgent need to enhance the diagnosis and management of MH through the utilization of relevant tools.

**Patient concerns:**

In this case, a 52-year-old woman underwent radical cervical cancer surgery under general anesthesia, with no family or significant medical history. She experienced a gradual increase in end-tidal carbon dioxide (ETCO_2_) to a maximum of 75 mm Hg and a rise in body temperature from 36.5 to 37.5 °C in a very short period, as well as a blood gas analysis showing a pH of 7.217.

**Diagnosis:**

The anesthesiologist immediately used The WeChat applet-based National Remote Emergency System for Malignant Hyperthermia (MH-NRES), and the score was 40, which indicated that the patient was very likely to have MH.

**Interventions:**

We immediately discontinued sevoflurane and switched total intravenous anesthesia to maintain general anesthesia, with a rapid intravenous infusion of dantrolene sodium.

**Outcomes:**

The ETCO2 and the temperature quickly dropped to normal, followed by successful completion of the surgery, and the patient was discharged 8 days after surgery.

**Lessons:**

The experience can provide a basis use of MH-NRES and improve the ability of anesthesiologists to deal with intraoperative MH as well as increase the survival probability of patients.

## 1. Introduction

Malignant hyperthermia (MH) is a rare autosomal dominant skeletal muscle hypermetabolic syndrome. The incidence of MH in patients undergoing general anesthesia ranges from 1 in 10,000 to 1 in 150,000. It is observed more frequently in children (1 in 15,000) compared to adults (1 in 50,000), and the incidence in men is approximately twice as high as that in women.^[1–3]^ The prevalence of MH can be as high as 1 in 400 individuals with genetic abnormalities. Currently, ryanodine receptor-1 (RYR1), calcium voltage-gated channel subunit alpha 1s (CACNA1S), and SH3 and cysteine-rich domain 3 (STAC3) are associated with MH.^[4]^ Variations within the RYR1 gene are responsible for the majority of MH cases.^[5]^ Patients with a genetic predisposition to malignant MH exhibit hypermetabolic reactions when exposed to certain volatile inhalation anesthetics such as halothane, sevoflurane, desflurane, isoflurane, as well as depolarizing muscle relaxants like succinylcholine. Additionally, MH can also be triggered by factors such as strenuous exercise and exposure to high temperatures.^[5]^

Anesthesiologists usually utilize the Clinical Grading Scale (CGS) for rapid diagnosis of MH. However, CGS is complex and encompasses multiple criteria. The caffeine–halothane contracture test is the golden standard for MH diagnosis, but it is not immediately diagnostic and is not applicable during acute MH episodes. In addition, although the immediate relatives of patients usually undergo MH screening by genetic testing, but false-negative results are frequent. Given that MH is a rare condition, anesthesiologists often lack experience in timely management based on CGS. Moreover, the cost of storing dantrolene sodium, the specific antidote for MH, is very high. Especially in China, due to its vast territory and large population, there are regional differences in the allocation of medical resources among provinces, resulting in imbalances. Consequently, establishing an integrated first-aid system for MH with Chinese characteristics has become a practical challenge for Chinese anesthesiologists.

## 2. Detailed case description

The patient, a 52-year-old female weighing 62 kg, was diagnosed with stage IB2 cervical squamous cell carcinoma. The planned surgical under general anesthesia, included transabdominal extensive hysterectomy, bilateral salpingo-oophorectomy, pelvic lymph node dissection, and para-aortic lymph node dissection. Upon entering the room, the vital signs were stable. The blood pressure was 118/79 mm Hg, heart rate was 61 bpm, oxygen saturation was 99%, and body temperature was 36.5 °C. The patient and her family members had never been exposed to general anesthesia. All preoperative laboratory examination indices were within the normal range.

**Figure 1.**
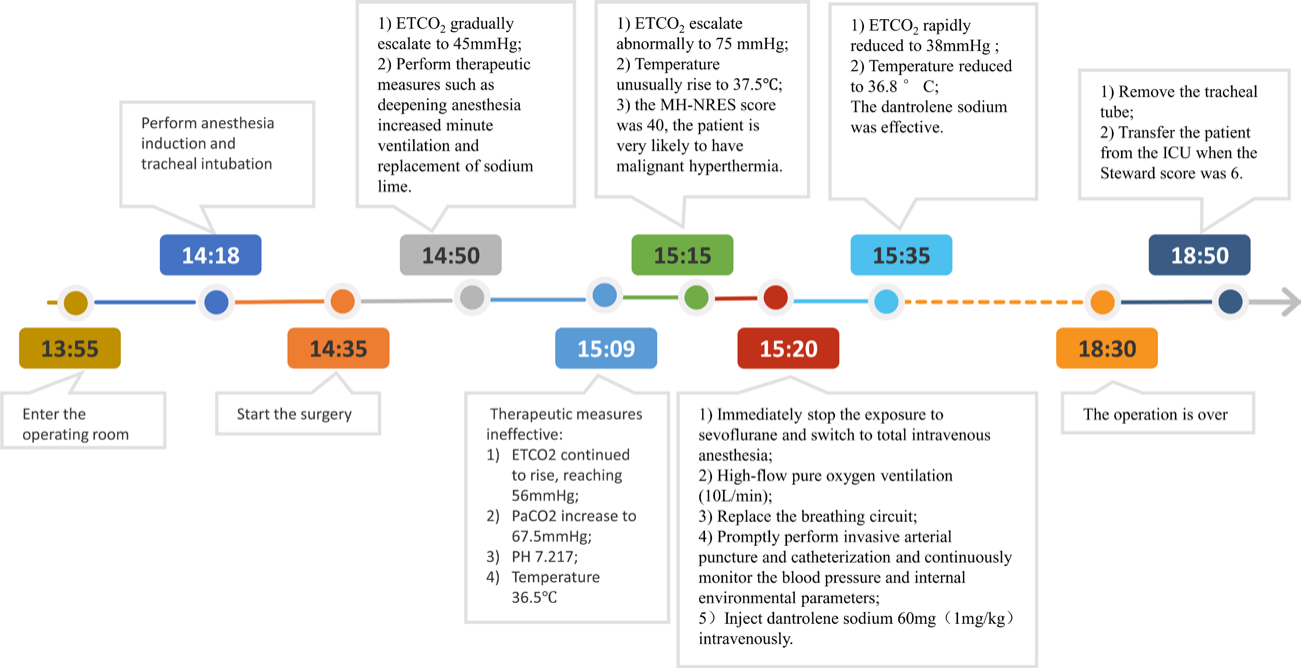
Timeline of the case presentation.

The whole surgical procedure is shown in Figure 1. At 14:18, the routine anesthesia induction was administered with the following formulation: 2 mg midazolam, 20 μg sufentanil, 7 mg cisatracurium, and 80 mg propofol. The vital signs remained stable throughout anesthesia induction, and tracheal intubation was successfully performed. Inhale 3% sevoflurane to maintain anesthesia. Additionally, dexmedetomidine hydrochloride was also continuously infused at a rate of 0.4 μg/kg/h via a pump.

The surgical procedure began at 14:35 after anesthesia. Monitoring indicators first changed at 14:50, with the end-tidal carbon dioxide (ETCO_2_) gradually rising from 35 to 45 mm Hg. Furthermore, the heart rate escalated from 60 to 95 bpm, and the blood pressure increased from 95/68 to 130/90 mm Hg. Meanwhile, the surgeon reported tense abdominal muscles and inadequate visibility of the surgical field. A supplementary dosage of 8 mg cisatracurium was administered to induce muscle relaxation, along with 5 μg sufentanil for pain relief. Concurrently, recalibrate minute ventilation immediately in response to rising ETCO_2_ levels. The tidal volume was set to 450 mL, respiratory rate to 16 breaths per minute (bpm), and average airway pressure to 14 cm H_2_O, meanwhile, we replaced the sodium lime. However, even with these adjustments, the patient’s ETCO_2_ continued to rise, reaching 56 mm Hg. The patient’s temperature was monitored using a nasopharyngeal probe, which displayed a normal body temperature of 36.5 °C. The blood gas analysis showed a partial pressure of carbon dioxide (PaCO_2_) of 67.5 mm Hg, pH of 7.217, and HCO_3_ of 27.4 mmol/L. No other significant abnormalities were observed. Subsequently, the patient’s body temperature began to gradually increase, accompanied by a sudden and substantial surge in ETCO_2_ levels. The maximum recorded values for body temperature and ETCO_2_ were 37.5 °C and 75 mm Hg.

**Figure 2.**
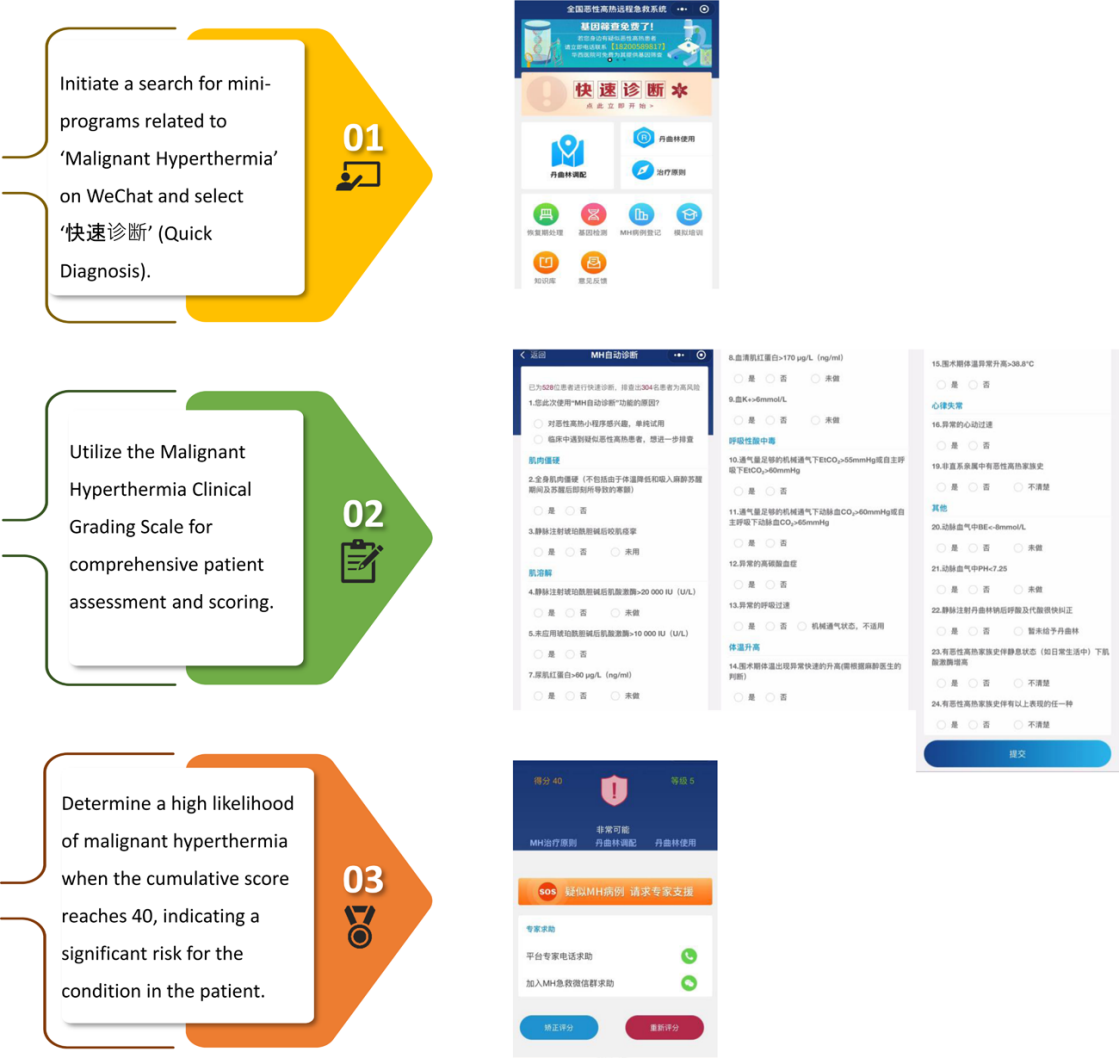
The process of using MH-NRES.

The WeChat applet-based National Remote Emergency System for Malignant Hyperthermia (MH-NRES) was promptly employed for rapid diagnosis (Fig. 2). The MH-NRES showed that the patient’s score was determined to be 40 points (Supplementary Table 1, http://links.lww.com/MD/M37). According to the scoring system, a score of 40 points indicates almost certain MH. In accordance with the guidelines outlined in the “Guidelines for Malignant Hyperthermia 2020” published by the British Society of Anesthesiologists, an immediate rescue plan was promptly implemented.^[6]^ First, we withdrew the patient from sevoflurane and switched to a continuous infusion of propofol at a rate of 500 mg/h to maintain intravenous general anesthesia. In addition, we intermittently administered cisatracurium and sufentanil. Simultaneously, we initiated high-flow pure oxygen ventilation at a rate of 10 L/min and replaced the breathing circuit. We promptly performed invasive arterial puncture and catheterization, and continuously monitored the blood pressure and internal environmental parameters. In addition, we rapidly injected dantrolene sodium at a dose of 1 mg/kg intravenously (LiZhu, China, Approval No. Guoyaozhunzi H20203530). Approximately 5 minutes following the dantrolene sodium, the patient’s ETCO_2_ levels rapidly decreased to 38 mm Hg, and the body temperature dropped to 36.8 °C. Throughout the procedure, the patient’s vital signs remained stable, and no significant abnormalities were found. The entire surgery operation was completed quickly and lasted for 3 hours and 55 minutes. During the procedure, the patient encountered a blood loss of 300 mL. The total crystalloid fluid infusion amounted to 3000 mL, and the cumulative urine output amounted to 650 mL (Table 1). The urine color was a faint shade of yellow. Subsequently, the patient was extubated successfully and transferred to the intensive care unit (ICU) for close monitoring.

**Table 1:** Intraoperative parameters of the patient.

Characteristic	Value
Maximum HR (bpm)	101
Minimum HR (bpm)	52
Maximum body temperature (°C)	37.5
Maximum ETCO_2_ (mm Hg)	75
Maximum PaCO_2_ (mm Hg)	67.5
Minimum PH	7.217
Bloody loss (mL)	300
Total fluids intake (mL)	3000
Total urine output (mL)	650

ETCO_2_ = end-tidal carbon dioxide; HR = heart rate; PaCO_2_ = partial pressure of carbon dioxide; PH = potential hydrogen.

Notably, there was no recurrence of MH observed. On the 3rd day postoperation, the patient was transferred from the ICU. Subsequently, on the 8th day postoperation, the patient was discharged from the hospital. During the 1-month follow-up period, no significant complications or issues were observed. Genetic sequencing of blood samples revealed suspicious mutations at sites c.12587T>C and c.6313T>C in the RYR1 gene. These mutations were preliminarily determined to have unknown clinical significance.

## 3. Discussion

MH is characterized by a significantly high mortality rate and rapid deterioration, necessitating timely and appropriate treatment. Mechanically ventilated patients with MH typically exhibit elevated ETCO_2_ levels, while individuals with spontaneous breathing may experience an increase in respiratory rate and elevated ETCO_2_ levels.^[6]^ MH commonly occurs following the administration of inhalation anesthetics and depolarizing muscle relaxants during surgery, with a certain latency period. For example, a patient undergoing coronary artery bypass surgery was diagnosed with MH 72 minutes after being transferred to the ICU due to a sudden rise in body temperature and ETCO_2_ levels.^[7]^ Consequently, effectively managing the perioperative period following the use of triggering agents is critical.

Although rare, MH is extremely lethal. From 1985 to October 2020, there were 136 MH cases in China, with a mortality rate of 55.9%.^[8,9]^ The rapid deterioration poses a significant challenge for clinicians in terms of prompt diagnosis. The rarity of MH limits the familiarity of anesthesiologists with this disease, making timely diagnosis and appropriate treatment difficult to achieve. Therefore, it is urgent to establish and enhance the domestic MH management system. Fortunately, the advent of smart mobile devices has ushered in a new era of mobile healthcare. The continuous miniaturization and powerful computing capabilities of these devices offer valuable assistance in the diagnosis of acute diseases and the management of chronic conditions.^[8]^ In this case, we present the development initiated by West China Hospital of Sichuan University on July 22, 2022-MH-NRES. The utilization of MH-NRES during surgeries enables real-time diagnosis and treatment recommendations, while also incorporating an online simulation training mode that empowers anesthesiologists to enhance their diagnostic and management skills related to MH through self-study.

Dantrolene sodium, the sole specific medication for MH, has obtained approval from the United States (US) Food and Drug Administration. However, this drug is burdened with high storage costs and poses significant challenges in routine preparation. Moreover, due to the uneven distribution of medical resources among provinces in China, it is impractical for all medical institutions in China that administer general anesthesia to consistently provide dantrolene sodium.^[10]^ Notably, the MH-NRES system serves multiple purposes. First, it aids anesthesiologists in promptly diagnosing and actively managing MH. Also, it offers free genetic screening and case registration for suspected MH patients and individuals susceptible to MH. Additionally, the system facilitates real-time monitoring and management of dantrolene sodium stockpiles. Furthermore, the system has implemented an emergency mechanism to swiftly distribute dantrolene sodium to regional medical institutions, addressing the issues of drug unavailability and inadequate preparation in certain medical facilities. Essentially, MH-NRES compiles comprehensive information from medical institutions across the country that have reserved dantrolene sodium. By organizing and categorizing this proximity information, the system enables rapid dissemination of information between regional medical institutions. This ensures that medical institutions lacking dantrolene sodium can swiftly acquire the necessary drug supplies in cases of malignant hyperthermia. Given its substantial contributions, the nationwide promotion of the MH-NRES system is highly warranted.

In this case report, the patient exhibited an elevation in ETCO_2_ levels and a gradual increase in body temperature following a 30-minute exposure to sevoflurane. Despite enhanced ventilation and administration of muscle relaxants, these symptoms persisted. After excluding other potential factors, suspicion of an MH attack arose. Although the caffeine–halothane contracture test (CHCT) is currently considered the “gold standard” for diagnosing MH, it is primarily conducted in research laboratories in China and has limited application in clinical practice. Consequently, the CGS scale is often utilized for the initial clinical assessment of suspected MH cases. In this particular case, the MH-NRES system (accessible by searching for “malignant hyperthermia” in Chinese on WeChat) was employed, which helped us quickly obtain the CGS score. The patient obtained a score of 40, indicating she is very likely to have malignant hyperthermia. The MH-NRES system is a compact program based on the WeChat application, which is widely used by the Chinese population. Anesthesiologists can conveniently access this program through WeChat without the need for additional downloads that would occupy storage space on their phones. Moreover, the system is user-friendly and offers comprehensive functionality, effectively aiding anesthesiologists in managing MH during emergency situations.^[11]^

It is worth noting that genetic testing revealed a mutation in the RYR1 gene in the patient, specifically at the mutation sites c.12587T>C and c.6313T>C. RYR1 serves as the primary calcium release channel in the sarcoplasmic reticulum, playing a pivotal role in skeletal and cardiac muscle cells. Mutations in the RYR1 gene are closely linked to the MH development. The mutation in the RYR1 gene disrupts the normal function of the RYR1 channel, resulting in dysregulation of calcium ion release within the sarcoplasmic reticulum. In 2022, Tan et al analyzed the genetic testing results of 4 Chinese MH patients and identified 2 RYR1 mutations (c.5317C>T and c.12587T>C) and 1 CACNA1S mutation (c.5399T>C) in the same patient.^[9]^ Therefore, we postulate that the mutation within the RYR1 gene may represent a potential pathogenic locus for MH. In individuals with MH, exposure to triggers such as inhalational anesthetics or depolarizing muscle relaxants causes abnormal RYR1 channels to release excessive calcium ions, resulting in severe muscle rigidity and hypermetabolism, ultimately leading to MH. For suspected MH patients with RYR1 gene mutations, it is crucial to avoid using inhalational anesthetics and depolarizing muscle relaxants as triggers and closely monitor the patient’s vital signs during surgery. Further research on RYR1 gene variations can enhance our understanding of MH pathogenesis and facilitate the development of novel treatment approaches.

## 4. Conclusions

MH is a severe complication associated with anesthetic drugs. The development of MH-NRES has made significant contributions to the rapid diagnosis and proactive management of MH cases by anesthesiologists, as well as their active involvement in the entire process of MH management. Additionally, it triggers an emergency deployment mechanism for the timely allocation of dantrolene sodium in regional medical centers, this initiative effectively resolves the challenges of drug unavailability and insufficient preparedness encountered by certain healthcare institutions. Consequently, it provides a practical adjunctive diagnostic and therapeutic tool for the national malignant hyperthermia emergency system.

## 5. Patient perspective

I am a cervical cancer patient with no family or significant medical history. During the radical operation for cervical cancer, the author demonstrated great concern for my well-being. When my monitoring indicators changed, the anesthesiologist promptly utilized MH-NRES for diagnosis and treatment. Thanks to their timely intervention, my vital signs stabilized, and I successfully completed the surgery. I am grateful for the author’s expertise in managing this situation.

## 6. Patient anonymity and informed consent

The patients understand that personal medical information that may be included in case reports will be redacted or anonymized to protect their privacy. The patient consented to the publication of this case report and hopes that by sharing his experience, it will provide the medical community with useful information and lessons to improve the diagnosis and treatment of other patients.

## Author contributions

**Conceptualization:** Yang Xiao, Rou Yu, Juan Gu.

**Data curation:** Yang Xiao.

**Formal analysis:** Yang Xiao.

**Investigation:** Yang Xiao.

**Methodology:** Yang Xiao, Rou Yu, Juan Gu.

**Validation:** Juan Gu.

**Writing—original draft**: Yang Xiao.

**Writing—review & editing:** Juan Gu.

## Supplementary Material


