# Supplementary material for: Diagnosis and rescue of malignant hyperthermia induced by anesthesia during radical surgery in a cervical cancer patient using the National Remote Emergency System: A case report
Source: Medicine (Baltimore). 2024 Apr 19;103(16):e37699. doi: 10.1097/MD.0000000000037699 (PMC11030021; doi:10.1097/MD.0000000000037699)
Supplement: Supplementary file 1 [file medi-103-e37699-s001.docx]

| Process | Indicator | Point | #Yes or No or Not Done or Not Clear | Score |
| --- | --- | --- | --- | --- |
| Rigidity | Generalized muscular rigidity (in absence of shivering due to hypothermia, or during or immediately following emergence from inhalational general anesthesia) | 15 | Not Clear | 0 |
|  | Masseter spasm shortly following succinylcholine administration | 15 | Not Done | 0 |
| Muscle Breakdown | Elevated creatine kinase>20,000 IU after anesthetic that included succinylcholine | 15 | Not Done | 0 |
|  | Elevated creatine kinase >10,000 IU after anesthetic without succinylcholine | 15 | Not Done | 0 |
|  | Cola colored urine in perioperative period | 10 | Not Done | 0 |
|  | Myoglobin in urine >60 ug/L | 5 | Not Done | 0 |
|  | Myoglobin in serum >170 ug/L | 5 | Not Done | 0 |
|  | Blood/plasma/serum K^+^>6 mmol/L (in absence of renal failure) | 3 | Not Done | 0 |
| Respiratory Acidosis | ETCO_2_>55 mmHg with appropriately controlled ventilation | 15 | Not Done | 0 |
|  | Arterial PaCO_2_>60mmHg with appropriately controlled ventilation | 15 | Not Done | 0 |
|  | ETCO_2_>60 mmHg with spontaneous ventilation | 15 | Not Done | 0 |
|  | Arterial PaCO_2_>65 mmHg with spontaneous ventilation | 15 | Not Done | 0 |
|  | Inappropriate hypercarbia (in anesthesiologist's judgment) | 15 | Yes | 15 |
|  | Inappropriate tachypnea | 10 | Not Done | 0 |
| Temperature Increase | Inappropriately rapid increase in temperature (in anesthesiologist's judgment) | 15 | Yes | 15 |
|  | Inappropriately increased temperature >38.8℃ in the perioperative period (in anesthesiologist's judgment) | 10 | No | 0 |
| Cardiac Involvement | Inappropriate sinus tachycardia | 3 | No | 0 |
|  | Ventricular tachycardia or ventricular fibrillation | 3 | No | 0 |
| Family History (used to determine MH susceptibility only) | Positive MH family history in relative of first degree | 15 | Not Clear | 0 |
|  | Positive MH family history in relative not of first degree | 5 | Not Clear | 0 |
| Other indicators that are not part of a single process | Arterial base excess more negative than -8mmol/L | 10 | No | 0 |
|  | Arterial pH <7.25 | 10 | Yes | 10 |
|  | Rapid reversal of MH signs of metabolic and/or respiratory acidosis with iv dantrolene | 5 | Not Done | 0 |
|  | Positive MH family history together with another indicator from the patient's own anesthetic experience other than elevated resting serum creatine kinase | 10 | Not Clear | 0 |
|  | Resting elevated serum creatine kinase (in patient with a family history of MH) | 10 | Not Clear | 0 |
| Total score |  |  |  | 40 |

#YES：the Score= Point; NO and Not Done and Not Clear: the Score=Zero

| Raw Score Range | Description of likelihood |
| --- | --- |
| 0 | Almost Never |
| 3-9 | Unlikely |
| 10-19 | Somewhat less than likely |
| 20-34 | Somewhat greater than likely |
| 35-49 | Very likely |
| 50+ | Almost certain |

From：Larach MG, Localio AR, Allen GC, et al. A clinical grading scale to predict malignant hyperthermia susceptibility. Anesthesiology. 1994;80(4):771-779.
